# The role of the neutrophil and formed elements of the blood in an *in vitro* model of reperfusion injury

**DOI:** 10.1155/S0962935193000122

**Published:** 1993

**Authors:** John A. Barrett, Claudia K. Derian, Robert S. Swillo, Richard F. Woltmann, Mark H. Perrone

**Affiliations:** 1 Department of Cardiovascular Biology Rhône-Poulenc Rorer Central Research Horsham PA USA; 2 RW Johnson Pharmaceutical Research Institute Welsh and McKean Roads Spring House PA 19477 USA

## Abstract

Using The globally ischaemic isolated guinea-pig heart we conducted studies to assess the role of activated neutrophils (PMNs) and the role of the endothelium in reperfusion injury. Reperfusion injury was induced by a 20 min period of global ischaemia followed by a 30 min reperfusion with Krebs' buffer supplemented with f-Met–Leu–Phe (fMLP) and heparinized blood. Ischaemia alone or blood alone resulted in a complete recovery in contractile function measured by developed pressure, fMLP (500 μM) and blood, administered to normoxic hearts did not affect contractile function. The combination of 100 μM fMLP and blood beginning at reperfusion and continuing for 30 min decreased the recovery in contractile function (max. 33 ± 6% reovery) while buffer and 100 pM fMLP resulted in a complete recovery in function. In hearts infused with buffer and neutropenic blood incubated with 100 μM fMLP a complete recovery in function was observed. Isolated peritoneal neutrophils, 7–70 × 10^5^ PMN/ min, incubated with 100 μM fMLP and Krebs' solution decreased contractile function in a concentration-related manner (max. 44 ± 11% recovery). Platelets, plasma or red blood cells alone incubated with fMLP did not decrease recovery in developed pressure. Platelets and PMN incubated with 100 μM fMLP did not, while red blood cells and PMN did, elicit a reduction in recovery in contractile function (34 ± 4% recovery). A 20 min period of global ischaemia destroys the functional integrity of the endothelium (response to Ach). Pre-treatment of the heart with sufficient H_2_O_2_ to functionally damage the endothelium, followed by infusion of Krebs' solution supplemented with blood and 100 μM fMLP also elicited a reduction in recovery of contractile function (42 ± 15% recovery). In summary, partially activated neutrophils play a major role in reperfusion injury and there exists a cooperativity between the RBC and PMN in this model.


Mediators of Inflammation, 2, 85–92 (1993)

### The role of the neutrophil and formed elements of the blood in an *in vitro* model of reperfusion injury

John A. Barrett,[CA,*] Claudia K. Derian, Robert S. Swillo, Richard F. Woltmann and Mark H. Perrone

Department of Cardiovascular Biology, Rhône-Poulenc Rorer Central Research, Horsham, PA, USA

*Present Address:* RW Johnson Pharmaceutical Research Institute, Welsh and McKean Roads, Spring House, PA 19477, USA

[CA] Corresponding Author

**Using the globally ischaemic isolated guinea-pig heart we conducted studies to assess the role of activated neutrophils (PMNs) and the role of the endothelium in reperfusion injury. Reperfusion injury was induced by a 20 min period of global ischaemia followed by a 30 min reperfusion with Krebs' buffer supplemented with f-Met–Leu–Phe (fMLP) and heparinized blood. Ischaemia alone or blood alone resulted in a complete recovery in contractile function measured by developed pressure. fMLP (500 μM) and blood, administered to normoxic hearts did not affect contractile function. The combination of 100 μM fMLP and blood beginning at reperfusion and continuing for 30 min decreased the recovery in contractile function (max. 33 ± 6% reovery) while buffer and 100 μM fMLP resulted in a complete recovery in function. In hearts infused with buffer and neutropenic blood incubated with 100 μM fMLP a complete recovery in function was observed. Isolated peritoneal neutrophils, 7–70 × $10^5$ PMN/min, incubated with 100 μM fMLP and Krebs' solution decreased contractile function in a concentration-related manner (max. 44 ± 11% recovery). Platelets, plasma or red blood cells alone incubated with fMLP did not decrease recovery in developed pressure. Platelets and PMN incubated with 100 μM fMLP did not, while red blood cells and PMN did, elicit a reduction in recovery in contractile function (34 ± 4% recovery). A 20 min period of global ischaemia destroys the functional integrity of the endothelium (response to Ach). Pre-treatment of the heart with sufficient $H_2O_2$ to functionally damage the endothelium, followed by infusion of Krebs' solution supplemented with blood and 100 μM fMLP also elicited a reduction in recovery of contractile function (42 ± 15% recovery). In summary, partially activated neutrophils play a major role in reperfusion injury and there exists a cooperativity between the RBC and PMN in this model.**

**Key words:** Endothelium, fMLP, Neutrophil, Platelet, Red blood cell, Reperfusion injury

### Introduction

The restoration of blood flow to the ischaemic myocardium via angioplasty, coronary bypass or fibrinolytic therapy has been shown to limit myocardial infarction and was thought to preserve ventricular function.[1] However, at reperfusion the introduction of oxygen, cellular elements and, particularly, the neutrophil (PMN) into the ischaemic myocardium elicits a deleterious cascade of events which limit ventricular function and myocardial salvage. This phenomenon is called reperfusion injury.[2–4] Under normal conditions, the PMNs move along the vascular wall, but do not adhere.[5–7] However, the restoration of flow (reoxygenation) after an ischaemic period has been shown to cause an accumulation of PMNs in the ischaemic myocardium suggesting their role in reperfusion injury.[4,8] PMN contains a number of substances capable of mediating vascular injury such as cationic proteins, acids, proteases which destroy vascular basement membranes, substances which induce release of $PGI_2$ from the endothelium and promote PMN adhesion and PMN-derived elastase which lyses endothelial cells. The removal of PMNs via mechanical[9] or physiological means[10–12] or inhibition of adhesion to the endothelium[13,14] has been shown to decrease myocardial damage and preserve ventricular function. The present study was performed to assess the interaction of the formed elements of the blood and the role of the endothelium in an *in vitro* model of reperfusion injury.

### Methods

Guinea-pigs of either sex (250–400 g) were anaesthetized with 35 mg/kg pentobarbital i.p. and

© 1993 Rapid Communications of Oxford Ltd administered heparin (150 U/kg i.p.) to inhibit microembolism in the coronary circulation. Hearts were quickly removed and mounted on a modified Langendorff apparatus and perfused with the Krebs–Henseleit buffer having a composition of 118.2 mM NaCl, 4.7 mM KCl, 2.2 mM $CaCl_2$, 1.2 mM $HPO_4$, 1.2 mM $MgSO_4$, 25 mM $NaHCO_3$ and 11 mM D-glucose and oxygenated with $O_2$–$CO_2$ (95%:5%) at 37°C. For assessment of developed pressure a fluid-filled balloon-tipped cannula was inserted into the left ventricle via the left atria and connected to a Gould pressure transducer (P23ID Gould, Oxnard, CA). After a 15 min equilibration period, end diastolic pressure was adjusted to 5 mmHg which was approximately 70–80% of the maximal stretch on the length pressure curve. Developed pressure was calculated as the difference between left ventricular systolic and diastolic pressures. Perfusion flow (Masterflex 7520, Barrington, IL) was adjusted to achieve a control mean perfusion pressure of 60 mmHg (approximately 10 ml/min). Therefore, a change in coronary resistance was detected by a change in perfusion pressure. Myocardial temperature was maintained at 37°C by placing the heart in a heated chamber. The effects on developed pressure, heart rate and perfusion pressure were continuously displayed on a strip chart recorder (Grass model 7D).

*Protocol:* On completion of a 15 min stabilization period, the preparation was rendered globally anoxic for 20 min by shutting off flow to the system while maintaining the temperature at 37°C. The hearts were then continuously reperfused with buffer (using the same flow as the control) and heparinized blood (0.1 ml/min) that had been pre-incubated with fMLP for 10 min at 37°C. The effects on contractility, heart rate and perfusion pressure (coronary vascular resistance) were monitored for 30 min post-reperfusion.

*Measurement of oxidative burst activity:* To determine the degree of neutrophil activation in response to fMLP stimulation, the following study was performed. Heparinized guinea-pig whole blood or blood diluted to a final concentration of 1% blood in 1 ml Krebs' buffer and warmed to 37°C was studied. Luminol was added to a final concentration of 5 $\mu$g/ml. Oxidative burst activity was measured in a continuously recording six-channel chemiluminometer (Biolumat LB9505, EG&G Berthold). After a 30 s stabilization period, fMLP was injected to induce the oxidative burst. The response was monitored for 5 min. The extent of the oxidative burst was quantified by integrating the area under the curve.

*Induction of neutropenia:* To assess the role of the neutrophil in this model a group of guinea-pigs were rendered neutropenic by combining the methods of Bogman *et al.*[15] and Henderson *et al.*[16] Briefly, the animals were treated with 1.75 mg/kg mechlorethamine hydrochloride (Sigma) i.p. on day one followed by 1 mg/kg, i.p. on days two and three. To prevent infection, the animals were kept in a laminar flow unit under aseptic conditions. On the fourth day, the animals were anaesthetized and arterial blood was collected in a heparinized syringe (Sarstedt monovette; Li-heparin, Germany). Differential leukocyte determinations and neutrophil counts were performed using a Unopette kit (test 5856, Becton-Dickinson, Rutherford, NJ) and a haemacytometer. Mechlorethamine treatment resulted in a marked reduction in leukocytes (9–15 $\times 10^6$ cells/ml control *vs.* $1 \times 10^6 \pm 0.09 \times 10^6$ cells/ml) with neutrophils preferentially suppressed by greater than 95% and mononuclear leukocytes by 50%.

*Isolation of peritoneal neutrophils:* To determine the role of the neutrophil and its interaction with the formed elements of the blood, the following isolations were performed. Guinea-pig peritoneal neutrophils were isolated as follows: briefly, 18 h prior to isolation, guinea-pigs were administered 6 ml of 6% Na-caseinate in saline i.p. (Sigma). Eighteen hours later, the guinea-pigs were euthanized and the cells recovered via peritoneal lavage. Viability (>98%) of the preparation was determined via trypan blue exclusion.

*Platelet isolation:* Platelets were isolated from citrated guinea-pig concentrates using a gel filtration technique previously described by Ruggeri *et al.*[17] The final platelet plug was resuspended in Krebs' solution to a final concentration of $2 \times 10^8$ cells/ml.

*Red blood cell isolation:* Citrated guinea-pig blood (0.38% citrate) was centrifuged at 120 × ***g*** for 15 min. The plasma was removed and the red blood cell pellet was washed three times with normal saline and centrifuged at 950 × ***g*** for 15 min. The pellet was then resuspended in sufficient Krebs' solution to achieve a haematocrit of 42.

*Endothelial function:* Since myocardial ischaemia followed by reperfusion results in endothelial dysfunction characterized by a decreased release of endothelium-derived relaxant factor, we wished to determine the role of the endothelium in this model using the following protocol: on completion of a 15 min stabilization period, the hearts were paced at 4–5 V DC at 10% above the threshold rate. Acetylcholine (ACh) (0.5 $\mu$M) was infused for 1 min to demonstrate endothelial functional integrity. When the preparation stabilized, 0.5 $\mu$M sodium nitroprusside (SNP) was then administered for 1 min to assess endothelium-independent vasodilatory effects. Pacing was then halted and the heart rendered globally ischaemic for a period of 20 min. After 10 min of reperfusion (when all parameters returned to pre-ischaemic baselines) pacing was reinstituted and ACh and SNP administered as stated above.

*$H_2O_2$ induced injury:* To determine the role of free radical-induced endothelial dysfunction, we performed the following protocol: on completion of a 15 min stabilization period, the hearts were paced at 4–5 V DC at 10% above the threshold rate. Acetylcholine (0.5 μM) was infused for 1 min to demonstrate endothelial functional integrity. When the preparation stabilized, 0.5 μM SNP was administered for 1 min to assess endothelial-independent vasodilatory effects. Pacing was halted and the preparation returned to control values. $H_2O_2$ (0.1 mM) was infused into the Krebs' solution for 20 min. The hearts were then paced, as above, and the ACh treatment repeated. After pacing was halted and the preparation returned to control values, blood incubated with 100 μM fMLP for 10 min was infused into the Krebs' solution and the effects on developed pressure, heart rate and perfusion pressure monitored.

*Statistics:* All data are expressed as the mean ± S.E. Data were analysed using analysis of variance and Dunnett's test or when appropriate Student's *t*-test. Differences were considered significant at $p < 0.05$.

### Results

Isolated hearts ($n = 6$) subjected to a 20 min period of ischaemia followed by a 30 min period of reperfusion did not have altered developed pressure, end diastolic pressure, heart rate or perfusion pressure values compared to the baseline control values of 59 ± 4 mmHg, 7 ± 2 mmHg, 218 ± 8 bpm and 61 ± 2 mmHg, respectively. Heparinized guinea-pig blood was infused, beginning at reperfusion and continuing for 30 min at 100 μl/min and resulted in a complete recovery in developed pressure, while heart rate and perfusion pressure were unchanged. fMLP in Krebs' solution, administered to hearts not rendered ischaemic ($n = 5$), at concentrations up to 500 μM did not alter developed pressure, end diastolic pressure, heart rate and perfusion pressure from the baseline values of 57 ± 5 mmHg, 6 ± 2 mmHg, 213 ± 5 bpm and 61 ± 2 mmHg, respectively. fMLP (30 and 100 μM) ($n = 6$ each) incubated in Krebs' solution for 10 min and infused at 100 μl/min for 30 min did not affect (98 ± 13 and 123 ± 22% recovery, respectively) recovery in contractile function. The combination of 3 and 10 μM fMLP ($n = 5$ each) incubated in blood for 10 min and administered at 100 μl/min, beginning at reperfusion and continuing for 30 min, did not affect (113 ± 15 and 102 ± 5% recovery, respectively) the recovery in contractile function, heart rate or perfusion pressure. Increasing concentrations of 30 and 100 μM fMLP ($n = 7$ each) significantly decreased the recovery in contractile function in a concentration-related manner (max. 33 ± 6% recovery), increased perfusion pressure (52 ± 8%) and did not affect heart rate. Increasing concentrations up to 300 μM did not further decrease contractile function or increase perfusion pressure. Heart rate remained unchanged (Table 1).

**Table 1.** Summary of the effects of various interventions in isolated guinea-pig hearts subjected to global ischaemia followed by reperfusion

| Treatment[a] | Control | | | | Reperfusion | | | |
|---|---|---|---|---|---|---|---|---|
| | Developed pressure (mmHg) | End diastolic pressure (mmHg) | Heart rate (bpm) | Perfusion pressure (mmHg) | Developed pressure (mmHg) | End diastolic pressure (mmHg) | Heart rate (bpm) | Perfusion pressure (mmHg) |
| Ischaemia alone | 59 ± 4 | 7 ± 2 | 218 ± 8 | 61 ± 2 | 59 ± 8 | 4 ± 3 | 227 ± 9 | 66 ± 6 |
| 1% blood | 63 ± 4 | 6 ± 3 | 215 ± 12 | 61 ± 1 | 67 ± 7 | 4 ± 2 | 220 ± 9 | 69 ± 5 |
| fMLP and buffer | | | | | | | | |
| 30 μM | 50 ± 11 | 6 ± 2 | 193 ± 11 | 63 ± 1 | 44 ± 6 | 6 ± 3 | 201 ± 10 | 70 ± 3 |
| 100 μM | 55 ± 6 | 4 ± 4 | 198 ± 15 | 60 ± 1 | 66 ± 9 | 2 ± 3 | 196 ± 13 | 73 ± 3[b] |
| 300 μM | 57 ± 8 | 6 ± 1 | 206 ± 11 | 62 ± 1 | 36 ± 9[b] | 15 ± 4[b] | 200 ± 8 | 84 ± 5[b] |
| fMLP and 1% blood | | | | | | | | |
| 3 μM | 54 ± 7 | 8 ± 3 | 214 ± 11 | 59 ± 1 | 61 ± 6 | 5 ± 2 | 208 ± 9 | 68 ± 7 |
| 10 μM | 56 ± 7 | 5 ± 2 | 217 ± 8 | 61 ± 1 | 58 ± 10 | 4 ± 3 | 219 ± 9 | 69 ± 2 |
| 30 μM | 59 ± 4 | 7 ± 3 | 220 ± 11 | 64 ± 1 | 31 ± 6[b] | 18 ± 7 | 225 ± 14 | 88 ± 7[b] |
| 100 μM | 58 ± 4 | 6 ± 2 | 203 ± 11 | 62 ± 1 | 17 ± 5[b] | 32 ± 6[b] | 192 ± 10 | 99 ± 6[b] |
| 300 μM | 54 ± 11 | 6 ± 3 | 197 ± 12 | 63 ± 2 | 11 ± 5[b] | 41 ± 12[b] | 203 ± 9 | 89 ± 9[b] |

[a] All values are expressed as the mean ± S.E.M. ($n$ = 5–6).
[b] Comparison of control *vs.* 30 min post-reperfusion using Student's paired *t*-test; $p < 0.05$.

*Measurement of oxidative burst activity:* To determine the degree of neutrophil activation in response to fMLP stimulation, we studied the effects of increasing concentrations of fMLP in both whole blood and in a 1% blood solution which corresponds to the final dilution at the level of the heart. fMLP at concentrations up to 100 $\mu$M failed to elicit an oxidative burst in guinea-pig heparinized whole blood. fMLP (0.3–10 $\mu$M) when administered to 1% guinea-pig blood elicited a concentration-related increase in the oxidative burst with the maximal amount of release observed at 3 $\mu$M. Interestingly, the 1 $\mu$M concentration of fMLP elicited approximately 60% of the maximal oxidative burst (Fig. 1).

*Role of the neutrophil (PMN):* In isolated hearts ($n = 6$) subjected to a 20-min period of ischaemia followed by reperfusion with Krebs' solution supplemented with neutropenic blood and 100 $\mu$M fMLP, no significant reduction in developed pressure (88 $\pm$ 16% recovery), end diastolic pressure, heart rate or perfusion pressure values from the control values of 63 $\pm$ 8 mmHg, 6 $\pm$ 2 mmHg, 245 $\pm$ 10 bpm and 61 $\pm$ 1 mmHg, respectively, were observed. To assess the role of the PMN in this model we studied the effects of isolated peritoneal neutrophils incubated with 100 $\mu$M fMLP and Krebs' solution infused at 100 $\mu$l/min at 7 (normal blood), 10, 30 and 70 $\times 10^5$ cells/min ($n = 6$ each), beginning at reperfusion and continuing for 30 min. Isolated peritoneal PMNs elicited a significant concentration-dependent decrease in recovery of contractile function (max. 44 $\pm$ 11% recovery at 70 $\times 10^5$ cells/min) from the baseline values of 62 $\pm$ 4 mmHg, 7 $\pm$ 2 mmHg, for developed pressure and end diastolic pressure,

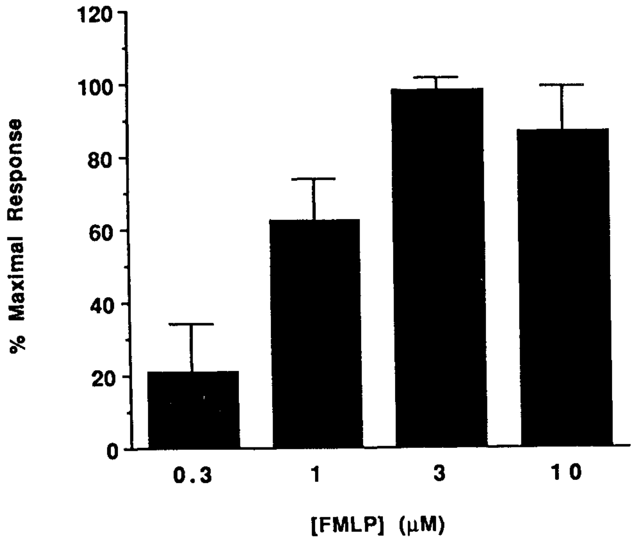

FIG. 1. Effects of increasing concentrations of fMLP on oxidative burst activity. fMLP dose-dependently enhanced the oxidative burst in 1% guinea-pig whole blood. The data represent the mean $\pm$ S.E. from two guinea-pigs assayed at each concentration of fMLP either in duplicate or triplicate. The maximal response was 7.8 $\times 10^4$ (4.1–13.0 $\times 10^4$) cpm.

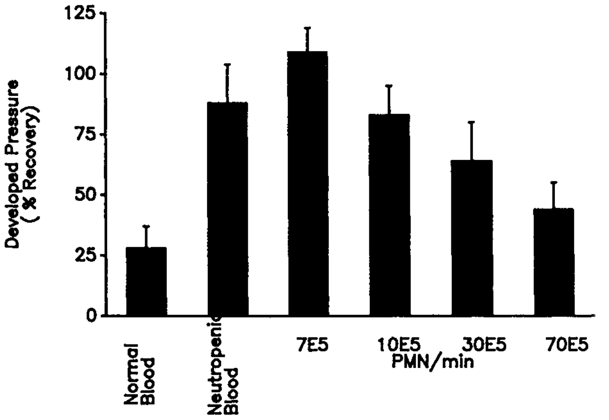

FIG. 2. Effects of blood and 100 $\mu$M fMLP, blood rendered neutropenic with nitrogen mustard and 100 $\mu$M fMLP, increasing concentrations of peritoneal PMN and 100 $\mu$M fMLP which were infused beginning at reperfusion and continuing for 30 min to isolated guinea-pig hearts rendered globally ischaemic for a period of 20 min. For the normal blood, neutropenic blood and 7 $\times 10^5$ PMN/min concentration, fMLP was incubated with the PMNs for 10 min and infused, beginning at reperfusion at a rate of 100 $\mu$l/min to isolated guinea-pig hearts rendered globally ischaemic for a period of 20 min. Each histogram depicts the mean $\pm$ S.E.% recovery in developed pressure at 30 min post-ischaemia for $n = 4$–6 hearts each. Concentrations of PMNs and fMLP are expressed as final tissue concentrations.

respectively (Fig. 2). Heart rate was unchanged and perfusion pressure tended to increase (34 $\pm$ 13%) from the baseline values of 203 $\pm$ 6 bpm and 60 $\pm$ 1 mmHg, respectively. Increasing the concentration of neutrophils above 70 $\times 10^5$ cells/min caused precipitation in the syringe.

*Interaction of formed elements:* To assess the role of the various blood components in this model, the following studies were conducted. In isolated hearts ($n = 5$) subjected to a 20 min period of ischaemia followed by reperfusion with platelets (2 $\times 10^7$ platelets/min) incubated with 100 $\mu$M fMLP for 10 min, and infused as a 1% platelet solution, recovery in developed pressure (84 $\pm$ 8% recovery) from the baseline value of 70 $\pm$ 5 mmHg was not altered. End diastolic pressure, heart rate and perfusion pressure were unchanged from the baseline values of 5 $\pm$ 2 mmHg, 221 $\pm$ 25 bpm and 62 $\pm$ 1 mmHg, respectively. In red blood cells, with the haematocrit adjusted to 42 with Krebs' solution and incubated with 100 $\mu$M fMLP for 10 min prior to infusion, ($n = 6$) recovery in developed pressure (109 $\pm$ 23% recovery) was also not affected. Developed pressure, end diastolic pressure, heart rate and perfusion pressure were unchanged from the baseline values of 55 $\pm$ 3 mmHg, 5 $\pm$ 2 mmHg, 229 $\pm$ 10 bpm and 61 $\pm$ 1 mmHg, respectively. Guinea-pig plasma incubated with 100 $\mu$M fMLP also did not diminish the recovery in developed pressure (82 $\pm$ 8% recovery). Developed pressure, end diastolic pressure, heart rate and perfusion pressure values were unchanged from the baseline values of 71 $\pm$ 5 mmHg, 5 $\pm$ 2 mmHg, 205 $\pm$ 15 bpm and 64 $\pm$ 2 mmHg, respectively (Fig. 3a).

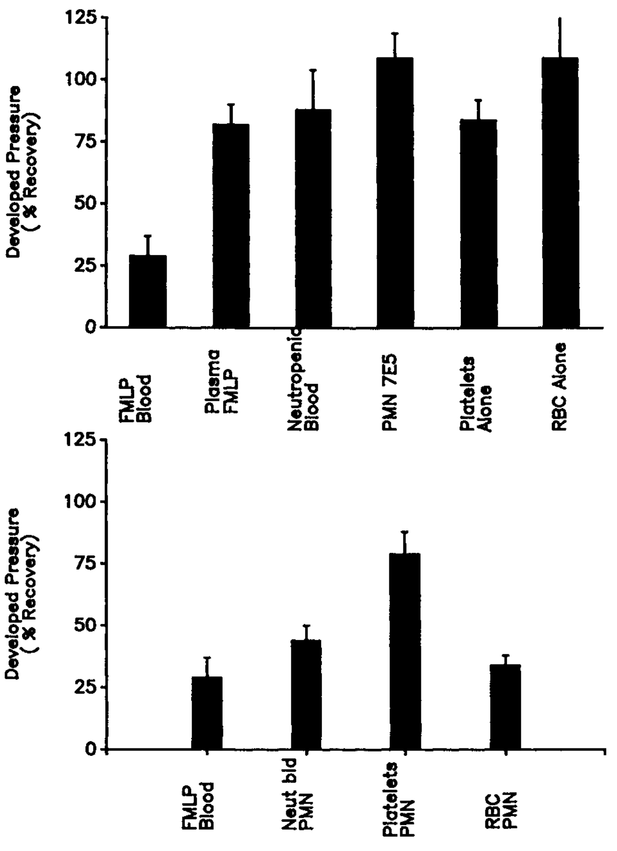

FIG. 3. (a) Effects of blood and 100 $\mu$M fMLP, blood rendered neutropenic with nitrogen mustard and 100 $\mu$M fMLP, peritoneal neutrophils (PMNs) ($7 \times 10^5$ PMN/min) and fMLP, blood plasma and fMLP, platelets ($2 \times 10^7$ platelet/min) and 100 $\mu$M fMLP and red blood cells (RBC) (haematocrit adjusted to 42) and 100 $\mu$M fMLP. (b) Effects of blood and 100 $\mu$M fMLP, neutropenic blood and peritoneal PMNs ($7 \times 10^5$ PMN/min) and 100 $\mu$M fMLP, platelets ($2 \times 10^7$ platelet/min) and peritoneal PMNs ($7 \times 10^5$ PMN/min) and 100 $\mu$M fMLP or red blood cells (RBC) (haematocrit adjusted to 45) and peritoneal PMNs ($7 \times 10^5$ PMN/min) and 100 $\mu$M fMLP incubated for 10 min at 37°C and infused beginning at reperfusion at a rate of 100 $\mu$l/min to isolated guinea-pig hearts rendered globally ischaemic for a period of 20 min. Each histogram depicts the mean $\pm$ S.E.% recovery in developed pressure at 30 min for $n = 4$–6 hearts each. Concentrations of fMLP, PMN and platelets are expressed as final tissue concentrations.

Neutropenic blood supplemented with peritoneal PMN sufficient to achieve the normal leukocyte count of $7 \times 10^6$ when incubated with 100 $\mu$M fMLP for 10 min and infused as a 1% solution ($n = 6$), beginning at reperfusion, resulted in a significant reduction in recovery in function ($44 \pm 6$% recovery). Developed pressure was decreased from $81 \pm 8$ to $25 \pm 6$ mmHg while end diastolic pressure was increased from $6 \pm 2$ to $38 \pm 7$ mmHg. Heart rate and perfusion pressure were not significantly altered from the baseline values of $208 \pm 11$ bpm and $62 \pm 1$ mmHg, respectively. Platelets and PMNs incubated with 100 $\mu$M fMLP for 10 min ($n = 8$) failed to significantly diminish the recovery in developed pressure ($79 \pm 9$% recovery) from the baseline value of $59 \pm 4$ mmHg. End diastolic pressure, heart rate and perfusion pressure were unchanged from their respective baseline values of $6 \pm 2$ mmHg, $189 \pm 3$ and $63 \pm 1$ mmHg. RBC and PMN incubated with 100 $\mu$M fMLP for 10 min and infused as a 1% solution ($n = 6$), elicited a significant reduction in the recovery in developed pressure ($34 \pm 4$% recovery) (Fig. 3b). Developed pressure was decreased from $61 \pm 4$ to $23 \pm 3$ mmHg while end diastolic pressure was increased from $6 \pm 1$ to $36 \pm 8$ mmHg. Perfusion pressure was increased $55 \pm 12$% from the baseline value of $63 \pm 1$ to $97 \pm 7$ mmHg while heart rate was unchanged from the baseline value of $216 \pm 12$ bpm.

*Role of the endothelium:* To assess the role of the endothelium the following two studies were conducted. In the first study, the functional integrity of the endothelium was assessed after a 20-min period of ischaemia. In the second study, the hearts were not rendered ischaemic, but the endothelium was functionally impaired via treatment with $H_2O_2$. In the first study, 0.5 $\mu$M ACh and 0.5 $\mu$M SNP administered to paced hearts (225 bpm) ($n = 6$) prior to a 20 min period of global ischaemia, significantly decreased perfusion pressure from the baseline value of $60 \pm 1$ mmHg by $21 \pm 6$% and $25 \pm 4$%, while developed pressure and end diastolic pressure were unchanged from the baseline values of $59 \pm 5$ and $5 \pm 2$ mmHg, respectively. After a 10-min period of reperfusion (developed pressure returned to baseline $52 \pm 8$ mmHg), 0.5 $\mu$M ACh failed to elicit the vasodilator response, while the response to SNP was unchanged from the baseline value of $64 \pm 4$ mmHg.

In the second study, 0.5 $\mu$M ACh and 0.5 $\mu$M SNP administered to paced hearts prior to $H_2O_2$ treatment significantly decreased perfusion pressure from the baseline value of $60 \pm 1$ mmHg by $24 \pm 1$% and $26 \pm 3$%, respectively, while developed pressure and end diastolic pressure were unchanged from the baseline values of $60 \pm 3$ and $6 \pm 2$ mmHg, respectively. $H_2O_2$ (0.1 mM) for 20 min did not affect any of the parameters studied. Ach (0.5 $\mu$M) administered post-$H_2O_2$ failed to elicit the vasodilator response ($61 \pm 3$ to $64 \pm 5$ mmHg) while the SNP response was unchanged ($-21 \pm 4$%). When the preparation stabilized (approximately 15 min) the hearts were assigned to one of the treatment groups. Blood alone or 100 $\mu$M fMLP alone ($n = 6$ each), infused as a 1% solution for 30 min, did not affect any of the parameters studied. In contrast, 100 $\mu$M fMLP and blood significantly decreased developed pressure ($42 \pm 15$% recovery) (Fig. 4), while perfusion pressure increased by $30 \pm 11$% and heart rate was unchanged. Baseline values for developed pressure, end diastolic pressure and perfusion pressure were $71 \pm 7$, $6 \pm 3$ and $61 \pm 1$ mmHg, respectively.

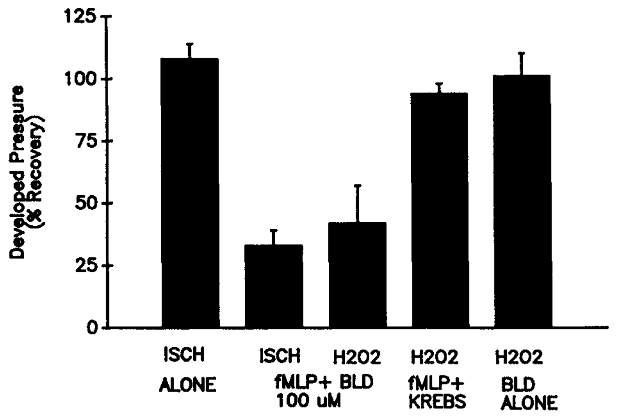

FIG. 4. Effects of 100 $\mu$M fMLP and blood, fMLP alone and blood alone in isolated guinea-pig hearts with functionally impaired endothelium. Endothelial damage was induced by 0.1 mM $H_2O_2$ pre-treatment for 20 min. Endothelial functional viability was determined by infusion of 0.5 $\mu$M acetylcholine prior to and after $H_2O_2$ treatment. Each histogram is the mean ± S.E. for $n = 4$–6 experiments.

### Discussion

The present study demonstrates that the neutrophil plays an essential role in an *in vitro* model of reperfusion injury. Secondly, it shows that neutrophils cooperate with other formed elements of the blood to exacerbate reperfusion injury. The isolated guinea-pig model utilized in the present study meets all the necessary criteria required to study this phenomenon. Since the ischaemic insult by itself does not decrease recovery in myocardial contractility, the chemoactivator, fMLP (100 $\mu$M), as well as blood alone, did not affect recovery in contractile function when administered to previously ischaemic hearts. However, after ischaemia the combination of fMLP and blood elicited a marked reduction in developed pressure indicating that some blood-borne element, when activated by fMLP, causes an extension of myocardial damage. Mullane and coworkers[18] using a bioassay technique demonstrated that previously infarcted rabbit hearts when stimulated with fMLP release $LTD_4$ and $LTB_4$. $LTB_4$ is a potent chemotactic agent that has been shown to amplify imflammatory responses and provoke PMN adhesion and degranulation. PMN degranulation results in further damage of the myocardium and increased PMN infiltration.[19,20]

The neutrophil appears to be one of the major causative components of reperfusion injury. Numerous studies have shown that the PMN levels markedly rise in the heart when it is subjected to a period of coronary occlusion followed by reperfusion.[2,8,9] The PMN when fully activated (sufficient to allow degranulation) releases a variety of substances such as proteolytic enzymes, oxygen-derived free radicals, as well as substances which facilitate further activation and recruitment of circulating PMN to the injured tissue.[2,4,21] In addition, the removal of the PMN from the preparation whether by antibody,[13,14] chemical[11,12] or mechanical[9] means has been shown to enhance recovery of function and lessen myocardial damage. In addition, inhibition of PMN adhesion to the damaged tissue has been shown to be effective.[22,23] In the present study, neutropenic blood stimulated with fMLP also prevented reperfusion-induced myocardial dysfunction. However, many of these manoeuvres which reduce neutrophil count also decrease other formed elements of the blood such as platelets and monocytes.[11,15] To determine if the other formed elements of the blood were responsible for the reperfusion-induced damage we administered neutropenic blood supplemented with peritoneal PMN sufficient to restore neutrophil count to normal. We found that the neutropenic blood supplemented with peritoneal PMN elicited a comparable reduction in contractile function as was observed when normal blood was used. These data therefore strongly suggest that the neutrophil is the major causative factor in reperfusion injury.

Interestingly, when the concentration of PMN present in normal blood was incubated with fMLP and administered at reperfusion, a complete recovery in developed pressure was observed. These data strongly suggest that there exists a cooperation between the neutrophil and other formed elements of the blood. Similar effects have been observed by others.[24,25] To test this hypothesis, we studied the effects of platelets alone and red blood cells alone incubated with fMLP. RBC or platelets alone failed to diminish recovery in developed pressure. However, the combination of RBC and neutrophils in the pressure of fMLP elicited a marked reduction in the recovery in contractile function which was of a similar magnitude to that observed when fMLP and normal blood were infused. These data suggest that there exists a cooperation between the RBC and PMN to enhance reperfusion-induced damage. This finding is supported by the work of McGee and Fitzpatrick[24] who demonstrated a transcellular biosynthesis of $LTB_4$ from erythrocyte–neutrophil interactions. These data may help to explain the paradoxical presence of $LTA_4$ hydrolase in the RBC while it lacks the machinery for the production of $LTA_4$. Interestingly, $LTA_4$ hydrolase is the rate-limiting enzyme in the formation of $LTB_4$ in the neutrophil. When the enzyme becomes saturated $LTA_4$ leaks out of the neutrophil and is picked up by the RBC which converts $LTA_4$ to $LTB_4$ resulting in additional PMN recruitment and subsequent degranulation.[24] In addition, the exists an extracellular transformation of $LTA_4$ to $LTB_4$. This extracellular transformation is sensitive to both heat and subject to proteolysis.[26]

Platelets have also been shown to accumulate in the reperfused myocardium in a pattern similar to that of the neutrophil. In addition, PMN depletion using an antineutrophil serum has also been shown to reduce platelet deposition in the reperfused myocardium.[21,27] Several investigators have demonstrated an interaction between platelets and PMN in arachidonic acid metabolism.[25,28,29] They determined that platelet-derived arachidonic acid can be converted by the neutrophil to $LTB_4$. Mueller *et al.*[29] demonstrated that isolated rabbit peritoneal PMN incubated with A23187 synthesized and released PAF. We postulated that stimulation of the guinea-pig PMN with fMLP would, when the PMN degranulated, release PAF and stimulate platelets to produce mediators which would result in a synergy between the platelet and PMN. The present data does not support this hypothesis, since the combination of platelets and PMN incubated with fMLP did not appreciably decrease contractile function ($80 \pm 9\%$ recovery). However, these data do not preclude an interaction between the platelet and PMN and may reflect the use of fMLP as an agonist. Coeffier *et al.*[25] studied the interaction of the PMN and platelet using zymosan as a chemoactivator and found a cooperation between the platelets and the PMN.

Reperfusion injury, we feel, involves a sequential series of steps beginning with endothelial damage[30–33] followed by PMN rolling, activation firm adhesion and degranulation. To determine the role of endothelial damage in this model, we studied the effects of blood alone, fMLP alone and the combination of fMLP and blood in isolated hearts pre-treated with sufficient $H_2O_2$ to functionally impair the endothelium. We found that blood alone or fMLP alone did not alter developed pressure while the administration of fMLP and blood markedly decreased contractile function. The magnitude of reduction was similar to that observed when fMLP and blood was administered at reperfusion. In addition, a 20 min period of ischaemia followed by reperfusion with Krebs' solution also functionally damaged the endothelium to the same magnitude as that observed with $H_2O_2$ treatment. It has previously been shown that $H_2O_2$ is a molecular species involved in endothelial injury and reperfusion injury.[34,35] Lewis *et al.*[34], using human umbilical vein, demonstrated that $H_2O_2$ treatment induced endothelial cell damage concomitant with endothelial-dependent neutrophil adhesion. Interestingly, the time course of the $H_2O_2$-induced endothelial cell-dependent PMN adhesion was rapid having a short time to onset with the peak effect occurring within 20 min.

In summary, we have demonstrated that partially activated neutrophils play a major role in reperfusion injury. In addition, our data indicate that there exists a cooperativity between the RBC and PMN in this model. Lastly, these data support our working hypothesis that reperfusion injury is the result of an ischaemic insult causing a localized endothelial injury due to endothelial-derived oxygen-free radical production. This injury permits partially activated PMNs to adhere resulting in their release of proteolytic substances and chemoactivators causing exacerbation of the damage, as well as the recruitment of additional PMN to the already damaged tissue.

### References

1. Braunwald E. The aggressive treatment of acute myocardial infarction. *Circulation* 1985; **71**: 1087–1092.
2. Reimer KA, Murry CE, Richard VJ. The role of neutrophils and free radicals in the ischemic-reperfused heart: why the confusion and controversy. *J Mol Cell Cardiol* 1989; **21**: 1225–1239.
3. Chu A, Cobb FR. Reperfusion alters the relation between blood flow and the remaining myocardial infarction. *Circulation* 1989; **79**: 884–889.
4. Lucchesi BR. Myocardial ischemia, reperfusion and free radical injury. *Am J Cardiol* 1990; **65**: 141–231.
5. Harlan JM. Neutrophil-mediated vascular injury. *Acta Med Scand* 1988; **715** (Suppl): 123–129.
6. Bevilacqua MO, Poker JS, Mendrick DL, Cotras RS. Identification of an inducible endothelial leukocyte adhesion molecule ELAM-1. *Proc Natl Acad Sci USA* 1987; **84**: 9238–9242.
7. Lucchesi B, Werns SW, Fantone JC. The role of the neutrophil and free radicals in ischemic myocardial injury. *J Mol Cell Cardiol* 1989; **21**: 1241–1251.
8. Engler RL. Free radical and granulocyte-mediated injury during myocardial ischemia and reperfusion. *Am J Cardiol* 1989; **63**: 19E–23E.
9. Engler RL, Covell JW. Granulocytes cause reperfusion ventricular dysfunction after 15-minute ischemia in the dog. *Circ Res* 1987; **61**: 20–28.
10. Romson JL, Hook BG, Kunkel SL, Abrams GD, Lucchesi BR. Reduction of the extent of ischemic myocardial injury by neutrophil depletion in the dog. *Circulation* 1983; **67**: 1016–1023.
11. Freed MS, Needleman P, Dunkel CG. Role of invading leukocytes in enhanced atrial eicosanoid production following rabbit left ventricular myocardial infarction. *J Clin Invest* 1989; **83**: 205–212.
12. Lorgeril M, Basmadjian A, Lavallee M, Clement R, Millette D, Rousseau G, Latoue J. Influence of leukopenia on collateral flow, reperfusion flow, reflow ventricular fibrillation and infarct size in dogs. *Am Heart J* 1989; **117**: 523–532.
13. Anfors K, Lundberg C, Lindborn L, Lundberg K, Beaty PG, Harlan JM. A monoclonal antibody to membrane glycoprotein complex CD18 inhibits PMN accumulation and plasma leakage *in vivo*. *Blood* 1987; **69**: 338–340.
14. Simpson PJ, Todd RF, Fantone JC, Mickelson JK, Griffin JD, Lucchesi BR. Reduction of experimental canine reperfusion-injury by monoclonal antibody (anti MO1, anti CD11b) that inhibits leukocyte adhesion. *J Clin Invest* 1988; **81**: 624–629.
15. Bogman MJJT, Cornelissen IMHA, Berden JHM, Dejong J, Koene RAP. A comparative study of the total irradiation as a method of inducing granulocyte depletion in mice. *J Immunol Methods* 1984; **70**: 31–38.
16. Henderson DK, Hockey LJ, Vukalcia LJ, Edwards JE. Effect of immunosuppression on the development of experimental hematogenous candida endophthalmitis. *Infect Immun* 1980; **27**: 628–631.
17. Ruggeri ZM, Demarco L, Gatti L, Bader R, Montgomery RR. Platelets have more than one binding site for von Willebrand factor. *J. Clin Invest* 1983; **72**: 1–12.
18. Mullane K, Hatala MA, Kraemer R, Sessa W, Westlin W. Myocardial salvage induced by REV-5901: an inhibitor and antagonist of the leukotrienes. *J Cardiovasc Pharmacol* 1987; **10**: 398–406.
19. Bagchi D, Das DK, Engelman RM, Prasad MR, Submanain R. PMN as a potential source of free radicals in ischemic-reperfused myocardium. *Eur Heart J* 1990; **11**: 800–813.
20. Sasaki K, Ueno A, Katori M, Kikawada R. Detection of LTB4 in cardiac tissue and its role in infarct extension through leukocyte migration. *Cardiovasc Res* 1988; **22**: 142–148.
21. Mullane K, Westlin W, Kraemer R. Activated neutrophils release mediators that may contribute to myocardial injury and dysfunction associated with ischaemia and reperfusion. *Ann NY Acad Sci* 1988; **524**: 103–121.
22. Mileski WJ, Winn RK, Vedder NB, Pohlman TH, Harlan JM, Rice CL. Inhibition of CD-18 dependent adherence reduces organ injury after hemorrhagic shock in primates. *Surgery* 1990; **108**: 206–212.
23. Carden DL, Smith JL, Korthuis RJ. PMN mediated microvascular dysfunction in post ischemic canine skeletal muscle role of granulocyte adherence. *Circ Res* 1990; **66**: 1436–1444.
24. McGee JE, Fitzpatrick F. Erythrocyte–neutrophil interactions: formation of LTB4 by transcellular biosynthesis. *Proc Natl Acad Sci USA* 1986; **83**: 1349–1353.

25. Coeffier E, Delautier D, Lecouedic J, Chignard M, Deniyot Y, Benveniste J. Cooperation between platelets and neutrophils for PAF-acether formation. *J Leuk Biol* 1990; **47**: 234–243.
26. Fitzpatrick F, Haeggstrom J, Granstrom E, Samuelsson B. Metabolism of LTA4 by an enzyme in blood plasma: a possible leukotactic mechanism. *Proc Natl Acad Sci USA* 1983; **80**: 5425–5429.
27. Bednar M, Smith B, Pinto A, Mullane K. Neutrophil depletion suppresses In-labelled platelet accumulation in infarcted myocardium. *J Cardiovasc Pharmacol* 1985; **7**: 906–912.
28. Kuehl FA, Dougherty HW, Ham EA. Interactions between prostaglandins and leukotrienes. *Biochem Pharmacol* 1984; **33**: 1–9.
29. Mueller HW, Oflaherty JT, Wykle RL. Biosynthesis of platelet-activating factor in rabbit polymorphonuclear neutrophils. *J Biol Chem* 1983; **258**: 6213–6218.
30. Lefer AM, Aoki N. Leukocyte dependent and leukocyte independent mechanism of impairment of endothelial mediated vasodilation. *Blood Vess* 1990; **27**: 162–168.
31. Mehta JL, Nichols WW, Donnelly WH, Lawson DL, Saldeen TP. Impaired coronary vasodilator response to acetylcholine and bradykinin after occlusion-reperfusion. *Circ Res* 1989; **64**: 43–54.
32. Weyrich AS, Ma XL, Lefer AM. The role of L-arginine in ameliorating reperfusion injury after myocardial ischaemia in the cat. *Circulation* 1992; **86**: 279–288.
33. Quillen JE, Sellke FW, Brooks LA, Harrison DG. Ischemia-reperfusion impairs endothelium-dependent relaxation of coronary microvessels but does not affect large arteries. *Circulation* 1990; **82**: 586–594.
34. Lewis MS, Whatley RE, Cain P, McIntyre TM, Prescott SM, Zimmerman GA. Hydrogen peroxide stimulates the synthesis of PAF by endothelium and induces endothelial cell-dependent neutrophil adhesion. *J Clin Invest* 1988; **82**: 2045–2055.
35. Kraemer R, Seligmann B, Mullane KM. PMN reduces cardiac function *in vitro* by release of $H_2O_2$. *Am J Physiol* 1990; **258**: H1847–H1855.

**Received 29 October 1992;
accepted in revised form 14 December 1992**
